# Effects of a Curcumin/Silymarin/Yeast-Based Mycotoxin Detoxifier on Redox Status and Growth Performance of Weaned Piglets under Field Conditions

**DOI:** 10.3390/toxins16040168

**Published:** 2024-03-25

**Authors:** Vasileios G. Papatsiros, Georgios I. Papakonstantinou, Nikolaos Voulgarakis, Christos Eliopoulos, Christina Marouda, Eleftherios Meletis, Irene Valasi, Polychronis Kostoulas, Dimitrios Arapoglou, Insaf Riahi, Georgios Christodoulopoulos, Dimitra Psalla

**Affiliations:** 1 Clinic of Medicine, Faculty of Veterinary Medicine, University of Thessaly, 43100 Karditsa, Greece; vpapatsiros@vet.uth.gr (V.G.P.); nvoulgarakis@uth.gr (N.V.); 2 Institute of Technology of Agricultural Products, Hellenic Agricultural Organization-Demeter (HAO-Demeter), 14123 Athens, Greece; chris_eliopoulos@hotmail.com (C.E.); dimarap@yahoo.com (D.A.); 3 Laboratory of Pathology, School of Veterinary Medicine, Faculty of Health Sciences, Aristotle University of Thessaloniki, 54124 Thessaloniki, Greece; 4 Laboratory of Epidemiology & Artificial Intelligence, Faculty of Public Health, School of Health Sciences, University of Thessaly, Terma Mavromichali St., 43100 Karditsa, Greece; el.meletis@gmail.com (E.M.); pkost@uth.gr (P.K.); 5 Laboratory of Physiology, Faculty of Veterinary Medicine, University of Thessaly, 43100 Karditsa, Greece; evalasi@uth.gr; 6 Biōnte Animal Nutrition, 43100 Reus, Spain; insaf.riahi@bionte.com; 7 Department of Animal Science, Agricultural University of Athens, 75 Iera Odos Street, Votanikos, 11855 Athens, Greece; gc@aua.gr

**Keywords:** CARBs, curcumin, detoxifier, fumonisins, mycotoxin, pig, silymarin, TAC, TBARS, yeast

## Abstract

The aim of this in vivo study was to investigate the effects of a novel mycotoxin detoxifier whose formulation includes clay (bentonite and sepiolite), phytogenic feed additives (curcumin and silymarin) and postbiotics (yeast products) on the health, performance and redox status of weaned piglets under the dietary challenge of fumonisins (FUMs). The study was conducted in duplicate in the course of two independent trials on two different farms. One hundred and fifty (150) weaned piglets per trial farm were allocated into two separate groups: (a) T1 (control group): 75 weaned piglets received FUM-contaminated feed and (b) T2 (experimental group): 75 weaned piglets received FUM-contaminated feed with the mycotoxin-detoxifying agent from the day of weaning (28 days) until 70 days of age. Thiobarbituric acid reactive substances (TBARSs), protein carbonyls (CARBs) and the overall antioxidant capacity (TAC) were assessed in plasma as indicators of redox status at 45 and 70 days of age. Furthermore, mortality and performance parameters were recorded at 28, 45 and 70 days of age, while histopathological examination was performed at the end of the trial period (day 70). The results of the present study reveal the beneficial effects of supplementing a novel mycotoxin detoxifier in the diets of weaners, including improved redox status, potential hepatoprotective properties and enhanced growth performance.

## 1. Introduction

Mycotoxins, which are secondary metabolic products formed by filamentous fungi (molds), are present in food and feed worldwide [1,2]. Even in small quantities, they pose a risk to human and animal health [3]. Cereal grains, which are frequently used in swine feed, are heavily contaminated with mycotoxins and pose a significant risk to swine health [4]. The consumption of mycotoxins (mycotoxicosis) can induce acute or chronic diseases even at low levels [1,5,6], causing significant economic losses due to reduced productivity in pigs (e.g., immunosuppression, reduced growth performance). The mycotoxins most frequently found in animal feed are aflatoxins (AFs, aflatoxin B1-AFB1, aflatoxin B2-AFB2, aflatoxin G1-AFG1, aflatoxin G2-AFG2); ochratoxin A (OTA); trichothecenes: deoxynivalenol (DON), T-2 toxin (T-2) and HT-2 toxin (HT-2); fumonisins (FUMs); and zearalenone (ZEN) [1,3]. Fumonisins (FUMs), a group of mycotoxins, are produced by *Fusarium verticillioides* (Sacc.) and *Fusarium proliferatum* and are the major contaminants of corn and corn-based products [7,8]. The multiple toxicological effects of FUMs are mainly due to fumonisin B1 (FUM-B1), the most prevalent and toxic of the major congeners present, and occasionally co-occur with FUM-B2, FUM-B3 and FUM-B4 [9]. Contamination of corn with FUMs is of particular concern in the animal feed industry [10], as it can lead to reduced growth performance and damage to the liver, lungs, kidneys and various systems (immune, respiratory reproductive, digestive) [11,12,13,14].

The predominant method of preventing and controlling mycotoxins is to incorporate mycotoxin detoxifiers into feed additives [15,16,17,18,19]. Mycotoxin detoxifiers are focused on reducing mycotoxin toxicity affecting animal health [20,21,22,23]. There are two types of mycotoxin-detoxifying agents: (a) the mycotoxin binders, which adsorb mycotoxins in the gastrointestinal tract (GIT), promote the excretion of mycotoxin–binder complexes in feces and forestall the uptake of mycotoxins; and (b) the mycotoxin modifiers, which are bio-transforming agents whose action is based on the conversion of mycotoxins by microorganisms/enzymes into non-toxic or less toxic metabolites [24,25]. Commercial mycotoxin-detoxifying agents often contain extra clay (e.g., sepiolite), various natural components or nutrient sources (e.g., yeast products, fiber, nucleotides, enzymes, herbs). These natural components aim to improve absorbability, immunity and gut health as well as enhance detoxification properties and prevent oxidative stress [26,27] Silymarin, which is produced from *Silybum marianum* [28], has well-known hepatoprotective, antioxidant and anti-inflammatory properties [29,30,31]. Previous researchers pointed out the beneficial effects of silymarin on the performance and health of weaned piglets [32] and sows [33,34]. Curcumin is a natural polyphenol extracted from *Curcuma longa* and widely used in traditional medicine and as a food additive [35,36,37]. Several studies have demonstrated its antioxidant properties [38,39]. Studies in weaning and growing pigs with curcumin supplementation have reported beneficial effects on redox status, gut health and especially on the growth performance of piglets with intrauterine growth restriction (IUGR) [40,41,42,43]. Furthermore, recent studies have shown that the supplementation of yeast products in weaning diets has positive effects on the health, growth performance, immune function, redox status and gut health of the piglets [44,45,46,47]. However, according to our knowledge of the literature, there are only a few in vivo studies on the use of curcumin and silymarin in the diets of weaned piglets with mycotoxin contamination. Our trial aimed to evaluate, under field conditions, the consequences of a novel mycotoxin detoxifier on performance and redox status in weaned piglets, under the dietary challenge of FUMs.

## 2. Results

### 2.1. Quantification of Mycotoxins in the Feed

Laboratory examination for mycotoxins in the feed samples from the two test farms revealed the presence of multiple mycotoxins (FUM-B1, FUM-B2 and AFB1), as shown in Table 1.

### 2.2. Redox Biomarkers

Table 2 presents plasma thiobarbituric acid reactive substance (TBARS) levels (μmol/L plasma), protein carbonyl (CARB) levels (nmol/mg protein) and the total antioxidant capacity (TAC) levels (mmol DPPH/L plasma) in the same groups at different time points per trial farm, as well as among different groups on the same study day for each trial farm. The laboratory examination of the plasma of the weaned piglets revealed statistically significant differences between the control (T1) and experimental (T2) groups for all biomarker values (Table 2). Specifically, a statistically significant reduction in TBARS and CARB levels was found for both control (T1) and experimental (T2) groups between day 45 and day 70 per trial farm. As for the TAC content, this was found to be statistically significantly increased on Farm 1 for both the control (T1) and experimental (T2) groups at day 70, whereas on Farm 2, the latter variable recorded a statistically significant increase in the experimental (T2) group only at the end of the trial period. 

### 2.3. Mortality and Performance Parameters

Table 3 presents the mortality rates and performance parameters in weaned piglets per trial farm. Significant differences were found between groups T1 and T2 in their mortality rates on both experimental farms (Table 3).

Body weight (BW) at day 45 and 70 and the average daily weight gain (ADWG) at both time points were significantly higher in the T2 group than in the T1 group for both trial farms, and their values were also significantly higher at 70 days in comparison to 45 days in each group (Table 3).

Significant differences between groups T1 and T2 were only found in feed intake at the two experimental farms and in the age groups of the trial (Table 3).

### 2.4. Gross and Histopathological Lesions

The necropsy of piglets revealed no remarkable gross lesions in any euthanized piglets.

**Figure 1:**
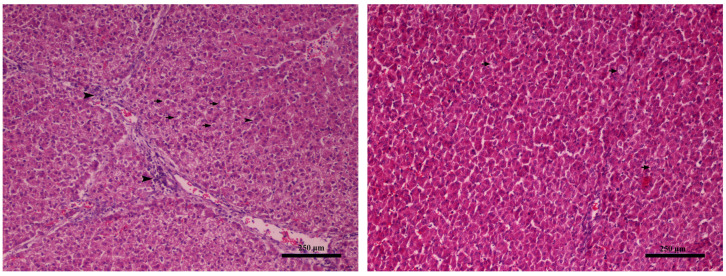
Liver (Farm 1, T1 control group piglet). **Left**: mild degeneration of hepatocytes seen as discoloration (black arrows) and mild periportal infiltration by lymphocytes and plasma (black arrowheads), magnification × 40. **Right**: without pathological changes, magnification × 40. Hematoxylin–eosin stain.

Upon histopathological examination, the liver specimens exhibited mild and non-specific lesions; however, an amelioration was evident in the respective control groups (Figure 1). Mild degeneration of hepatocytes (seen as discoloration) and mild periportal lymphoplasmacytic infiltration were observed in piglets from the control group T1 (Figure 1).

**Figure 2:**
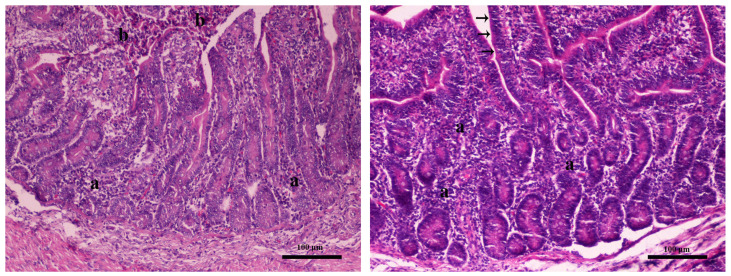
Intestine. **Left** (Farm 2, T1 control group piglet): mild infiltration of the lamina propria by lymphocytes and plasma cells (indicated as a) and necrosis of the epithelium (indicated as b), magnification × 100. **Right** (Farm 2, T2 experimental group piglet): mild infiltration of the lamina propria by lymphocytes and plasma cells (indicated as a) without necrosis—intact epithelium (black arrows), magnification × 100. Hematoxylin–eosin stain.

In intestine samples, necrosis of the epithelium and mild infiltration of the lamina propria by lymphocytes and plasma cells was noted in the control group T1 (Figure 2 left). Infiltration of the lamina propria by lymphocytes and plasma cells was noted in the target group, as well. However, the epithelium was rather intact, attributed to the strengthening of the mucosal barrier to surface bacteria (Figure 2 right).

**Figure 3:**
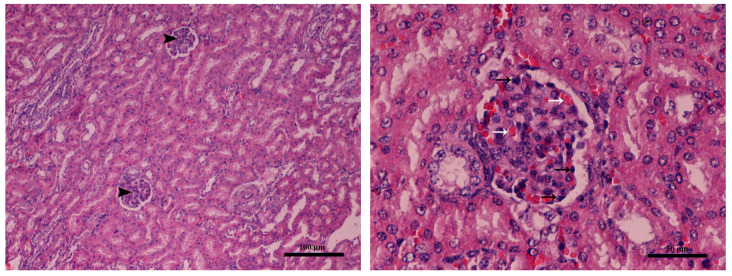
Kidney (Farm 1, T1 control group piglet): (**left,** magnification × 100 and **right**, magnification × 200) glomerulonephritis characterized by mild hypercellularity mononuclear (black arrows) and hyperemia of glomeruli (white arrows). Hematoxylin–eosin stain.

Mild glomerulonephritis was found in the kidneys of both groups (no differences were found between groups) (Figure 3).

No differences were found in the lymph nodes of either group. Mild hyperplastic lymphadenopathy was observed in a few cases, considered a non-specific reactive hyperplasia to common pathogens.

## 3. Discussion

The EU has announced maximum levels of mycotoxins in animal feed [48,49]. However, in most cases, the effects of contaminated feeds in the field are subclinical (e.g., reduced growth and reproductive performance, immunosuppression) due to lower levels of contamination [5]. To the best of the authors’ knowledge, this is the first field study to demonstrate that incorporating a multi-component mycotoxin-detoxifying agent (mix of bentonite, sepiolite, extracts of silymarin, curcumin and yeasts) into a FUM-contaminated diet has beneficial effects on the antioxidant status and growth performance of weaned piglets.

Mycotoxin contamination in animal feed has serious toxic effects, inducing severe negative health effects. These negative effects are due to oxidative stress and the increased number of free radicals, which induce damage to DNA, proteins and lipids [50,51,52]. Mycotoxins harm the antioxidant system of pigs, as they can cause an increase in the toxic by-product of lipid peroxidation, malondialdehyde (MDA), but also inhibit the activity of antioxidant defense mechanisms [53]. Indeed, in the current study, after 17 days of dietary challenge (from 28 to 45 days of age) with FUMs in weaned piglets, TBARS, a lipid peroxidation index, and CARB, a protein oxidation index, were detected at high levels. Remarkably, these findings were observed in both trial farms with differences in the level of FUM contamination. In agreement with our results, in a previous study performed in weaned piglets fed pure AFB1, increased TBARS and decreased TAC and growth performance were found [54]. According to another study, feeding corm contaminated with aflatoxins and FUMs did not affect either growth performance or MDA levels in nursery pigs [55]. However, in our study, TBARS and CARB levels were lower at 45 and 70 days of age in piglets of the experimental group (T2) compared to the control group (T1) at both time points. This finding ascertains the positive effect of a mycotoxin detoxifier on the redox status of weaned piglets. Notably, in both groups and at both farms, TBARS and CARB were reducing as development progressed; this may be attributed to the development of the antioxidant system in piglets after the stress of weaning [56]. On the other hand, the degree of reduction over time in both redox biomarkers was higher in the T2 group than in the T1 group, supporting the beneficial impact of the tested mycotoxin detoxifier on the antioxidant mechanisms of piglets. This effect was further demonstrated by the greater increase in TAC assayed in the T2 group over time. TAC is a biomarker that reflects the equilibrium between pro-oxidants and antioxidants in an animal’s bloodstream [57].

The degree of oxidative stress is related to several factors, including the ages of animals, the duration of mycotoxin co-contamination and their detected levels or synergistic effects [53]. Pigs fed with FUM-contaminated feed have reduced growth performance [8] and severe pathological changes in the liver [14], lungs [11], kidneys [12] and gastrointestinal structure [13]. In our study, the piglets fed with FUM-contaminated feed exhibited mild lesions in the liver, intestine and kidneys. However, the use of the tested mycotoxin detoxifier in weaning diets appeared to beneficially act on the histopathological structure of the liver, intestine and kidneys in the piglets. Moreover, FUM-B1 and AFB1 impaired the integrity of the jejunum monolayer-derived porcine IPEC (intestinal epithelial cell line) through alteration of its viability and reduction of TEER (transepithelial electrical resistance) [58]. 

Moreover, in our trial, increased levels of redox biomarkers (TBARS and CARB) were found in the plasma of weaned piglets that received FUM-contaminated feed, supporting the results of the aforementioned studies. Remarkably, the values of these redox biomarkers were increased in weaned piglets according to their age, as older piglets (70 days of age) had higher levels than younger ones. In addition, even if the level of AFB1 contamination on Farm 2 was below the EU recommendation, we noted that the redox biomarkers were raised in weaned piglets. This finding could be explained by the fact that these low levels of AFB1 can affect the redox status. According to a recent study, the exposure of weaned piglets to similar levels reduced the antioxidant capacity of the intestine and increased the production of pro-inflammatory cytokines [59].

The formulation of the mycotoxin detoxifier tested in the present study contains plant extracts of curcumin and silymarin with known antioxidant properties [35,36,37]. In addition, several in vitro studies have demonstrated their antioxidant effects on several mycotoxins, such as OTA, FUM-B1, ZEN and DON [60]. Nevertheless, our trial is the first study to evaluate the antioxidant effect of curcumin as a mycotoxin detoxifier in weaned piglets under field conditions with FUM-contaminated diets. Previous in vivo studies in poultry and rodents indicated significant hepatoprotective and antioxidant properties of silymarin against mycotoxins such as AFB1, and OTA [61,62]. The current study with the in vivo experiment in weaned piglets showed a significant decrease in redox biomarkers (TBARS and CARB) and an increase in TAC, confirming these previous reports. Our results are consistent with previous studies reporting that the use of detoxifiers with functional ingredients such as clay/inactivated yeast/botanicals/antioxidants improved their detoxification properties in weaned piglets exposed only to DON [63,64]. These studies reported improvements in growth performance, health status, immunity and gut health and reduced redox status [63,64]. In our previous trial with the addition of the same tested mycotoxin detoxifier in sows, positive effects on redox biomarkers, health and performance parameters were observed [65]. Our results on the anti-inflammatory and antioxidant effects of silymarin agree with the findings of previous studies in sows [33,34,66,67]. Nevertheless, our study is the first in vivo study with the use of curcumin and silymarin in weaned piglets under field conditions with FUM-contaminated diets. Previous ones have investigated the effects of curcumin in IUGR piglets only to a limited extent [40,42,68].

An additional outcome of the present study is the finding that, in weaned piglets, the tested mycotoxin detoxifier has positive effects on growth performance parameters, such as BW and ADWG at 45 and 70 days of age. Mycotoxins such as ZEN and FUMs have negative effects on the gut health and performance parameters in weaned piglets [8,69]. The relatively low intestinal absorption of FUMs in monogastric animals (only 1% to 6%) [70] suggests that the intestinal epithelium, which serves as a defense barrier and receiver of ingested contaminants, is exposed to high concentrations of ingested FUMs compared to other tissues [71]. Our results agree with the outcomes of previous studies reporting that the use of mycotoxin detoxifiers with functional components (e.g., clay, yeast, herbs) in weaned piglets treated with DON alone has beneficial effects on detoxification, growth performance, gut health and redox status [63,64]. Furthermore, our results support the findings of a recent study in weaned piglets under chronic FUM and AF exposure, demonstrating the beneficial effects of a mycotoxin detoxifier (containing bentonite and hydrolyzed yeast) on their growth performance [72]. Generally, it has been reported that postbiotics can prevent the negative effects of mycotoxin-co-contaminated feed on the growth and health parameters of weaned piglets [63]. Our results agree with recent studies that reported positive effects on the performance parameters of weaned piglets after the supplementation of yeast products in their diets [44,45,46,47]. The yeast cell wall extract contains the polysaccharide ß-D-glucan, which has been shown to have positive effects on performance and health parameters [63]. The yeast cell wall could, therefore, be useful for protection against mycotoxins, increasing nutrient absorption and decreasing the gathering of mycotoxins in various organs [63,73,74,75,76,77].

A high prevalence of mycotoxins is estimated in crops, especially in cereals that are the main feed ingredients in commercial pig farms [78,79]. This is a critical risk factor for the swine industry [80], and when cereals are contaminated, complete elimination of mycotoxins is not possible [16]. Szécsi et al. (2010) [81] reported that the proportions of FUM-B1, FUM-B2, FUM-B3 and FUM-B4 are typically 70–80%, 15–25%, 3–8% and 1–2%, respectively. Pigs are at the highest risk of contamination with FUMs because their feed is cereal-based. Today, commercial swine operations face multiple-mycotoxin toxicity rather than a single mycotoxin [64]. Certainly, the ideal way to prevent the negative effects of mycotoxins would be to avoid the feeding of mycotoxin-contaminated cereals. However, the use of mycotoxin-free feed is very difficult to achieve in practice. For this reason, multi-component mycotoxin detoxifiers are becoming increasingly necessary as an effective strategy for the prevention of mycotoxicosis in swine [64].

## 4. Conclusions

This field study has showed the positive effects on the redox balance and growth performance of weaned piglets when adding a novel mycotoxin detoxifier formulated with clays (bentonite and sepiolite), phytogenics (curcumin and silymarin) and postbiotics (yeast products). The weaned piglets in this field study were given feed contaminated with FUMs. The current results could serve as a starting point for future studies on the breakdown processes of the antioxidant properties of curcumin, silymarin and yeast products under laboratory conditions. Additional research is needed to investigate the effects of mycotoxin-contaminated feed and the benefits of regular supplementation with the tested mycotoxin detoxifier at various stages of production in pig farms.

## 5. Materials and Methods

### 5.1. Description of the Farms and Their Diets

Our trial was conducted between February 2023 and May 2023 at two commercial farrow-to-finish pig farms located in central Greece (Thessaly): Farm 1, where 600 sows of the Large White × Landrace (DanBred) breed were housed, and Farm 2, where 560 sows of the Large White × Landrace (Topigs Norsvin) breed were kept.

Both study farms had similar facilities. Each gilt/sow was individually identified and housed in a separate insemination station for artificial insemination with Duroc boar semen. One week before the expected farrowing date, the pregnant sows were moved from the mating area to farrowing pens, which were equipped with nipple drinkers and automatic feeders for sows and piglets. No feed was available in the farrowing pens. After weaning, the sows were returned to the mating area and kept individually in cages with slatted floors until the next insemination cycle.

Weaning took place at 28 days of age and then the piglets transitioned to the growth phase at 70 days of age. Prior to weaning, piglets of 18 days of age were vaccinated against *Mycoplasma hyopneumoniae* and *porcine circovirus type 2*. The sows underwent routine vaccinations for *porcine reproductive and respiratory syndrome type 1*, *suid herpesvirus 1*, *porcine influenza*, *porcine parvovirus 1*, *erysipelas*, *Escherichia coli*, *Clostridium perfringens type C*, *Clostridium novyi* and *Clostridium difficile*. In addition, the sows were treated with a single injection of ivermectin for parasite control 14 days before parturition.

Following the environmental stress model, all experimental animals were kept in identical stables with constant conditions (climate, ventilation, temperature and humidity). Drinking water was supplied automatically with nipple flow monitored daily by a technician. Routine monthly testing for chemical and microbiological factors was conducted and fully automated climate monitoring systems (Argos S, Microfan B.V., Nederweert, The Netherlands) were installed to regulate temperature and humidity in the barns.

The piglets had constant access to self-produced feed via connected automatic feeders. From the 7th to the 28th day, the day of weaning, the suckling piglets were offered creep feed pellets. The weaning diets, listed in Table 4, were in accordance with NRC recommendations [82] and consisted of grains such as corn, barley and soybeans. Special consideration was given to the diet of the control group, taking into account production aspects, before the treatment diet was introduced. The daily feeding order for all piglets in each pen was randomized. After weaning, the piglets were fed a commercial supplement containing premixes of essential vitamins, minerals, micro/macro elements and amino acids that met all requirements for a balanced diet according to current standards.

### 5.2. Quantification of Mycotoxins in Feed

Prior to commencing the study on the experimental farms, samples weighing 500 g of the final feed offered to weaned piglets were subjected to analysis and quantification for mycotoxins at both farms. The quantification process involved assessing eleven mycotoxins (AFB1, AFB2, AFG1, AFG2, FUM-B1, FUM-B2, OTA, ZEN, DON, T-2 and HT-2) utilizing the high-pressure liquid chromatography–mass spectrometry (HPLC–MS) detection methodology. These analyses were conducted at APSALAB (Andrés Pintaluba S.A.-APSA, Reus, Spain), following previously outlined procedures [65]. For the in vitro extraction of mycotoxins from feed samples, 5 g of the sample was placed into a centrifuge tube. Initially, the feed samples underwent grinding into fine powder using laboratory mill equipped with a sieve. Subsequently, 20 mL of extraction solution (comprising 80% acetonitrile–water solution with 0.1% formic acid) was added to the tube and agitated for 1 h. The resulting mixture was then centrifuged at 3500 rpm and 20 °C for 5 min, after which the liquid fraction was transferred to another tube for storage. An additional 20 mL of extraction solution was added to the remaining solid residue, shaken for a further 30 min and then subjected to centrifugation (3500 rpm, 20 °C, 5 min). The resulting 40 mL of extraction liquid underwent further centrifugation (3500 rpm, 20 °C for 5 min), from which 1 mL was transferred to an Eppendorf tube for subsequent centrifugation (12,500 rpm for 5 min). Finally, 80 µL of the solution was transferred to an HPLC vial and 20 µL of internal standard was added for injection into the device using the method outlined by Stroka et al. (2000) [83]. These mycotoxin standards were distinguished by C13 isotopes. The quantification of eleven mycotoxins (AFB1, AFB2, AFG1, AFG2, FUB1, FUB2, OTA, ZEN, DON, T-2 and HT-2) was carried out with the HPLC-MS technique, using a Zorbax RRHD Eclipse Plus C18 column (2.1 × 100, 1.8 µm) for analyte separation.

### 5.3. Experimental Material

This research investigated a novel mycotoxin-neutralizing agent (Biōnte^®^ Quimitōx^®^ Plus, developed by Biōnte Nutrition S.L., based in Reus-Tarragona, Spain). It contains a mixture of bentonite and sepiolite as well as phytogens (from natural extracts of silymarin and curcumin) and a combination of specific yeast extracts (including yeast cell wall and hydrolyzed yeast).

### 5.4. Experimental Design

#### 5.4.1. Study 1

One hundred and fifty (150) weaned piglets from a single batch were randomly allocated into two groups: (a) T1 (control group), comprising 75 piglets provided with FUM-contaminated feed (refer to Table 1), and (b) T2 group (experimental group), consisting of 75 piglets fed with FUM-contaminated feed (see Table 1) supplemented with 2.5 kg of the mycotoxin detoxifier (Biōnte^®^ Quimitōx^®^ Plus) until they reached 70 days of age.

#### 5.4.2. Study 2

One hundred and fifty (150) weaned piglets from a common batch were randomly allocated into two groups: (a) T1 (control group) comprising 75 piglets provided with FUM-contaminated feed (see Table 1), and (b) T2 group (experimental group) consisting of 75 piglets fed with FUM-contaminated feed (see Table 1) in addition to 2.5 kg of the evaluated mycotoxin detoxifier (Biōnte^®^ Quimitōx^®^ Plus) until they reached 70 days of age.

#### 5.4.3. Conduct of the Studies

Each experimental farm included 150 experimental piglets, divided equally into two experimental groups of 75 piglets each, which were housed in the same room in three pens of 25 piglets each. The individual piglets were identified with ear tags and the gender distribution was maintained at a ratio of 50/50 per pen. The piglets came from 15 litters per farm, ensuring that primiparous and multiparous sows were equally represented from parity 1 to 5, with four sows per parity. Littermates were evenly distributed between groups to equalize parity and average body weight. Weaned piglets were kept in an all-in, all-out batch production system and divided into experimental and control groups. Pens were color-coded according to group assignment to prevent physical interactions between piglets from different pens. During weaning, piglets were offered ZnO (2000 ppm) and amoxicillin (300 ppm) during the weaning phase to prevent post-weaning diarrhea. This supplement was administered one week before and one week after weaning, following the standard protocol of the experimental farms.

### 5.5. Blood Sampling

During the field trial, blood samples were taken from 36 weaned piglets per group. Twelve samples were collected from each group from three pens, resulting in a total of thirty-six samples. The samples were obtained from the external jugular vein at 45 and 70 days of age on both farms using a snout loop. The body weight and ear tags were recorded for each sample for identification purposes. BD Vacutainer^®^ plasma tubes (Becton Dickinson, Franklin Lakes, NJ, USA) with EDTA as the anticoagulant agent were used for blood collection. All blood samples were then centrifuged at 12,000× *g* for 10 min at 4 °C. Plasma was extracted, transferred to 1.5 mL microcentrifuge tubes and frozen at −80 °C for subsequent laboratory analysis.

### 5.6. Laboratory Examinations for Redox Biomarkers

The assessment of TBARS and CARB in plasma, which serve as redox biomarkers, was carried out according to the methodology described by Gerasopoulos et al. (2015) [84]. A modified version of the analysis by Keles et al. (2001) [85] was used to determine TBARS values, in which the TBARS concentration (μmol/L plasma) was calculated using the molar extinction coefficient of malondialdehyde (MDA). MDA reacts with thiobarbituric acid (TBA) in a ratio of 1:2 and forms a spectophotometrically measurable adduct. The molar coefficient of MDA was determined to be 155 × 103 M^−1^ cm^−1^. In addition, the method described by Patsoukis et al. (2004) [86], which is based on the molar extinction coefficient of 2,4-DNPH (22 × 103 M^−1^ cm^−1^), was used for the quantification of CARBS (nmol/mg protein). The determination of TAC (mmol DPPH/L plasma) in plasma was performed according to the method described by Janaszewska and Bartosz (2002) [87].

### 5.7. Histopathological Examination

The piglets euthanized at the end of the experiment underwent necropsy to assess possible macroscopic lesions on both trial farms. Six piglets per group (two per pen) were euthanized at 70 days of age on each trial farm by intravenous pentobarbital injection (Dolethal; 10 mg/kg) followed by exsanguination. For histopathological examination, tissue samples were taken from the lymph nodes, intestine, liver and kidneys. Subsequently, they were fixed in 10% buffered formalin for 48 to 72 h. Slides with 3–5 μm thick sections of formalin-fixed paraffin-embedded specimens (Paraplast Plus^®^, Kendall, UK) were then prepared and routinely stained with hematoxylin and eosin [88].

### 5.8. Mortality and Performance Parameters

In the clinical evaluation of weaned piglets, two main aspects were assessed: (a) mortality rate and (b) growth performance indicators. The body weight (BW; kg) of each piglet in two groups per experimental farm was measured three times: at 28 days of age (weaning), at 45 days of age and at 70 days of age. The analysis of average daily weight gain (ADWG; g/pig/day) was performed for two different experimental phases: (a) from 28 to 45 days of age and (b) from 45 to 70 days of age. The average daily weight gain during these intervals was calculated by subtracting the initial weight from the final weight and dividing by the duration of the respective production phase. In addition, the total feed intake (FI) and feed conversion ratio (FCR) were determined at 45 and 70 days of age. FI per animal was calculated as the total amount of feed in kg provided to each batch in each period, divided by the number of pigs in each pen. The FCR per animal was determined by dividing the total feed intake (kg) by the body weight (kg) during the study period. Data on deceased or removed piglets were included in the calculations.

### 5.9. Statistical Analysis

Each analysis was performed on three separate occasions. Results were expressed as mean ± standard deviation (±SD). The normal distribution of the data was assessed using the Kolmogorov–Smirnov and Shapiro–Wilk tests. To compare the control and experimental groups independently, the parametric data were assessed using the *t*-test for independent and paired samples, while the non-parametric data were analyzed using the Mann–Whitney test and the Wilcoxon test. Statistical significance was assumed at a 95% confidence level (*p* ≤ 0.05). All statistical analyses were performed using R programming language [89].

## Figures and Tables

**Table 1:** Detected mycotoxins in the feed of trial farms.

Detected Mycotoxin (µg/kg)	Farm 1	Farm 2	Maximum Level (µg/kg) *
Total FUMs	1220.3	2973.0	(FUM-B1 + FUM-B2) 5000
FUM-B1	970.2	2309.41	
FUM-B2	250.1	663.9	
AFB1	-	3.9	20
AFB2	<2.0	<2.0	
AFG1	<2.0	<2.0	
AFG2	<2.0	<2.0	
OTA	<4.0	<4.0	
ZEN	<12.0	<12.0	
DON	<40.0	<40.0	
T-2	<4.0	<4.0	
HT-2	<40.0	<40.0	

FUMs: fumonisins; FUM-B1: fumonisin B1; FUM-B2: fumonisin B2; AFB1: aflatoxin B1, AFB2: aflatoxin B2, AFG1: aflatoxin G1, AFG2: aflatoxin G2, OTA: ochratoxin A, ZEN: zearalenone, DON: deoxynivalenol, T-2: T-2 toxin, HT-2: HT-2 toxin. * European Commission (EC) 2006/576/EC [48] and 2002/32/EC [49].

**Table 2:** Levels (mean ± sd) of TBARSs (μmol/L plasma), CARBs (nmol/mg protein) and TAC (μmol DPPH/L plasma) in weaned piglets of control (T1) and experimental (T2) groups in study farms.

Farm 1 ^1^						
Parameters	Groups ^5^					
	Control (T1)			Experimental (T2)		
	Day 45	Day 70	*p* Value ^6^	Day 45	Day 70	*p* Value ^6^
TBARS ^2^	9.03 ± 1.09 ^a^	7.97 ± 0.61 ^b^	<0.001	8.48 ± 0.58 ^g^	6.07 ± 0.42 ^h^	<0.001
CARB ^3^	0.85 ± 0.08 ^c^	0.75 ± 0.12 ^d^	0.003	0.76 ± 0.09 ^i^	0.53 ± 0.12 ^j^	0.027
TAC ^4^	0.51 ± 0.06 ^e^	0.65 ± 0.10 ^f^	0.004	0.53 ± 0.06 ^k^	0.70 ± 0.03 ^l^	0.002
**(a,b,c,d,e,f,g,h,i,j,k,l): indicate statistical significance** (paired *t*-test, *p* ≤ 0.05).						
**Farm 2 ^1^**						
**Parameters**	**Groups ^5^**					
	**Control (T1)**			**Experimental (T2)**		
	**Day 45**	**Day 70**	***p* Value ^6^**	**Day 45**	**Day 70**	***p* Value ^6^**
TBARS ^2^	9.44 ± 0.52 ^a^	8.51 ± 0.51 ^b^	0.007	8.15 ± 0.56 ^e^	5.70 ± 0.96 ^f^	0.002
CARB ^3^	0.90 ± 0.11 ^c^	0.80 ± 0.06 ^d^	0.007	0.78 ± 0.08 ^g^	0.63 ± 0.04 ^h^	0.002
TAC ^4^	0.46 ± 0.02	0.51 ± 0.07	0.068	0.23 ± 0.03 ^i^	0.59 ± 0.06 ^j^	<0.001

^1^ This study was conducted independently on two different farms. The results are expressed as mean +/− SD, *n* = 75 per group. ^2^ TBARS (μmol/L plasma). ^3^ CARB (nmol/mg protein). ^4^ TAC (μmol DPPH/L plasma). ^5^ Different superscripts in the same row for each examined group indicate statistical significance (paired *t*-test, *p* ≤ 0.05). ^6^ The statistical analysis (paired *t*-test) was performed between days 45 and 70 separately for the control and the experimental groups.

**Table 3:** Mortality rates and performance parameters in weaned piglets of control (T1) and experimental (T2) groups in trial farms.

**Trial Period**	**Farm 1** ^**1**^			**Farm 2** ^**1**^		
	**Groups** ^**7**^			**Groups** ^**7**^		
	**T1 Group**	**T2 Group**	***p*** **Value** ^**8**^	**T1 Group**	**T2 Group**	***p*** **Value** ^**8**^
	**Mortality Rate** ^**2**^					
	8.0 (6/75)	4.0 (3/75)	<0.001	6.66 (5/75)	2.66 (2/75)	<0.001
**Body Weight (BW) ^3^**						
At weaning age	7.74 ± 0.68 ^a^	7.69 ± 0.72 ^a^	0.870	7.51 ± 0.55 ^a^	7.50 ± 0.63 ^a^	0.870
Day 45	14.86 ± 1.46 ^a^	17.28 ± 1.54 ^b^	<0.001	14.59 ± 1.25 ^a^	16.88 ± 1.14 ^b^	<0.001
Day 70	27.14 ± 1.64 ^a^	33.72 ± 1.92 ^b^	<0.001	26.96 ± 1.36 ^a^	31.44 ± 1.71 ^b^	<0.001
**Average Daily Weight Gain (ADWG) ^4^**						
Days 28–45	385.72 ± 21.28 ^a^	428.55 ± 2.22 ^b^	<0.001	378.86 ± 19.98 ^a^	421.38 ± 1.94 ^b^	<0.001
Days 45–70	586.48 ± 22.88 ^a^	657.83 ± 2.73 ^b^	<0.001	580.87 ± 22.49 ^a^	653.76 ± 2.26 ^b^	<0.001
**Feed Intake (FI) ^5^**						
Day 45	18.26 ± 1.31 ^a^	21.6 ± 1.23 ^c^	<0.001	18.21 ± 1.68 ^e^	21.17 ± 1.58 ^g^	<0.001
Day 70	40.82 ± 2.13 ^b^	50.1 ± 2.68 ^d^	<0.001	40.44 ± 2.2 ^f^	47.15 ± 2.62 ^h^	<0.001
**Feed Conversion Ratio ^6^**						
Day 45	1.25 ± 0.03 ^a^	1.25 ± 0.03 ^c^	0.45	1.25 ± 0.03 ^e^	1.25 ± 0.03 ^g^	0.31
Day 70	1.5 ± 0.03 ^b^	1.5 ± 0.03 ^d^	0.05	1.5 ± 0.03 ^f^	1.5 ± 0.03 ^h^	0.95

^1^ This study was conducted independently on two different farms. The results are expressed as mean +/− SD, *n* = 75 per group. ^2^ Mortality rate (%). ^3^ BW (kg). ^4^ ADWG (g/pig/day). ^5^ FI (kg). ^6^ Feed conversion ratio. ^7^ Different superscripts in the same row for each examined group indicate statistical significance (paired *t*-test, *p* ≤ 0.05). ^8^ The statistical analysis (paired *t*-test) was performed between days 45 and 70 separately for the control and the experimental groups. **(a,b,c,d,e,f,g,h): indicate statistical significance** (paired *t*-test, *p* ≤ 0.05).

**Table 4:** Dietary composition and nutritional elements found in the weaning feed across the experimental farms.

Composition of Ingredients (kg)	Farm 1		Farm 2	
	Age of Animals (Days)			
	28–45	45–70	28–45	45–70
Corn	440	450	506	506
Barley	260	200	50	50
Wheat bran	40	100	-	-
Soybean meal (46% crude protein)	140	145	200	200
Soybean oil	20	20	-	-
Protein concentrate (68% crude protein) *	40	25	40	40
Complementary feed with vitamin/mineral premix	-	-	200	200
Supplementary feed with vitamin/mineral premix	50	50	-	-
Mycotoxin binder	2.5	2.5	2.5	2.5
Tributyrin	-	-	1.0	1.0
Natural calcium carbonate	7.5	7.5	-	-
Total	1000	1000	1000	1000
**Analyzed nutrient components**	**Age of animals (days)**			
	**28–45**	**45–70**	**28–45**	**45–70**
Crude protein (%)	17.10	16.40	18.0	17.5
Crude fat (%)	4.80	4.60	3.0	3.0
Crude fiber (%)	3.10	3.30	3.0	3.3
Ash (%)	4.90	4.70	5.0	5.1
Lysine (%)	1.17	1.11	0.9	1.3
Methionine + Cystine (%)	0.72	0.70	0.62	0.60
Calcium (%)	0.69	0.61	0.9	0.8
Digestible phosphorus (%)	0.36	0.30	0.7	0.5
Sodium (%)	0.39	0.39	0.4	0.4

* Apsaprotein F68 (Andres Pintaluba SA, Reus, Spain).

## Data Availability

All data generated for this study are presented within the manuscript.

## Abbreviations

ADWG	Average Daily Weight Gain
AFB1	Aflatoxin B1
AFB2	Aflatoxin B2
AFG1	Aflatoxin G1
AFG2	Aflatoxin G2
AFs	Aflatoxins
BW	Body Weight
CARBs	Protein Carbonyls
DNPH	Dinitrophenylhydrazine
DON	Deoxynivalenol
FCR	Feed Conversion Ratio
FI	Total Feed Intake
FUM-B1	Fumonisin B1
FUM-B2	Fumonisin B2
FUM-B3	Fumonisin B3
FUM-B4	Fumonisin B4
FUMs	Fumonisins
GIT	Gastrointestinal Tract
HPLC-MS	High-Pressure Liquid Chromatography–Mass Spectrometry
HT-2	Trichothecene Toxin HT-2 toxin
MDA	Malondialdehyde
NRC	National Research Council
IPEC	Intestinal Epithelial Cell Line
IUGR	Intrauterine Growth Restriction
OTA	Ochratoxin A
SD	Standard Deviation
T-2	Trichothecene Toxin T-2 toxin
TAC	Total Antioxidant Capacity
TBARS	Thiobarbituric Acid Reactive Substance
TEER	Transepithelial Electrical Resistance
ZEN	Zearalenone
